# The Association between Polymorphisms of Vitamin D Metabolic-Related Genes and Vitamin D_3_ Supplementation in Type 2 Diabetic Patients

**DOI:** 10.1155/2019/8289741

**Published:** 2019-09-08

**Authors:** Zhiyong Hu, ShaSha Tao, Huaqing Liu, Guotao Pan, Bingyan Li, Zengli Zhang

**Affiliations:** ^1^Department of Occupational and Environmental Health, School of Public Health, Medical College of Soochow University, Suzhou, China; ^2^Lishui Center for Disease Control and Prevention, Lishui, China; ^3^Department of Nutrition and Food Hygiene, School of Public Health, Medical College of Soochow University, Suzhou, China

## Abstract

**Objective:**

To investigate the effect of single nucleotide polymorphisms (SNPs) of the key genes in vitamin D metabolic pathway on the serum 25(OH)D level after long-term vitamin D_3_ supplementation and provide a theoretical basis for rational vitamin D_3_ supplementation in diabetic patients with different genetic backgrounds.

**Methods:**

Patients with type 2 diabetes (T2DM) who met the inclusive criteria were given 800 IU of vitamin D_3_ daily for 30 consecutive months. Serum 25(OH)D levels was measured at enrollment and every 6 months after enrollment. The average value of four-time measurements represented individual serum 25(OH)D level during vitamin D_3_ supplementation. Multiplex TaqMan genotyping was used to determine the distribution of eight candidate SNPs in genes of DHCR7, CYP2R1, CYP27B1, CYP24A1, and VDR, which are key genes in the vitamin D metabolic pathway, in diabetic patients.

**Results:**

At baseline, the average serum 25(OH)D level was 22.71 ± 6.87 ng/mL, and 17.9% of patients had a ≥30 ng/mL level. During supplementation, the level of 25(OH)D increased significantly at each time point, and the average 25(OH)D level increased to 30.61 ± 5.04 ng/mL; however, there were 44.6% of patients whose serum 25(OH)D levels were still below 30 ng/mL. In the patients with CYP27B1 (rs10877012) G/T genotype, 71.79% achieved sufficient level of 25(OH)D, which was significantly higher than the other two genotypes (*P* < 0.05). Compared with those with T/T genotype, the RR of the patients with rs10877012 for <30 ng/mL level was 0.544 (95% CI: 0.291-0.917), and the RR after adjusting age and outdoor activity was 0.560 (95% CI: 0.292-0.970).

**Conclusion:**

The serum 25(OH)D level in patients with diabetes mellitus after long-term vitamin D_3_ supplementation is associated with CYP27B1 polymorphism. Patients with rs10877012 G/T allele have a better response to vitamin D_3_ supplementation.

**Trial Registration:**

This trial is registered with ChiCTR-IPC-17012657.

## 1. Introduction

Vitamin D has been found to be involved in a variety of public health-significant diseases including bone diseases [1], diabetes [2], cardiovascular diseases [3], cancer [4], and metabolic syndrome [4]. Due to its various extraosseous effects and the association between its deficiency and insulin resistance along with diabetes initiation, the serum vitamin D levels gain an extensive attention in the field of endocrinology [5]. A large number of cross-sectional studies have shown a negative correlation between vitamin D status and T2DM prevalence [2, 6]. While several longitudinal studies in Europeans [7], African-Americans [8], South Asians [9], and in China [10] still demonstrated low levels of serum 25(OH)D which could predict the risk of type 2 diabetes, vitamin D supplementation also reduced the incidence of diabetes accordingly [7, 10]. Consistent with this, there was evidence to show that vitamin D deficiency is significantly higher in diabetic patients than in normal population in China [11]. Recent studies find that vitamin D_3_ supplementation improved insulin secretion in diabetic patients [12] and show beneficial effects in diabetic patients with poor glycemic control [13]. Maintaining adequate vitamin D level in the population will be an important strategy to reduce the incidence of diabetes. Many physicians have accepted that vitamin D can be a component of prescription for diabetic patients. However, before that, there is still an important question, in view of the potential hazard of excessive vitamin D_3_ supplementation, what the level of vitamin D is sufficient for diabetic patients.

25(OH)D, the main circulating metabolite of vitamin D, is a biomarker to indicate the level of vitamin D in the body. The factors affecting circulating 25(OH)D level include sunshine exposure and dietary intake; however, its high heritability suggests that genetic factors also played important roles [14]. It is increasingly recognized that genetic factors influence serum 25(OH)D status. Previous studies based on European and Chinese twins and families have confirmed that genetic factors have a significant impact on the individual variation of 25(OH)D levels. The heritability is estimated to be as high as 53% [14, 15]. Although several rare Mendelian diseases can cause functional vitamin D deficiency [16–18], there are few studies to investigate the effects of common genetic variants on vitamin D status, especially in diabetic patients. If vitamin D status is associated with certain genotypes or SNPs, then some people may need higher or lower level of serum 25(OH)D than general level to minimize health risk. Whether individuals with genotypes known to influence the efficiency of vitamin D_3_ supplementation may require particular (“personalized”) recommendations with respect to optimizing vitamin D_3_ supplementation in order to minimize adverse health outcomes.

According to previous candidate gene studies [19] and genome-wide association studies [20, 21], some of the common SNPs in vitamin D metabolic pathway genes are found to be associated with the level of circulating 25(OH)D in common status. These SNPs encode key metabolic enzymes include 25-hydroxylase (CYP2R1), 1-hydroxylase (CYP27B1), 24-hydroxylase (CYP24A1), 7-dehydrocholesterol reductase (DHCR7), and vitamin D receptor (VDR). CYP2R1 converts vitamin D to 25(OH)D. CYP27B1 activates 25(OH)D to 1-alpha,25-dihydroxy-cholecalcifero (1,25(OH)_2_D_3_). CYP24A1 inactivates 25(OH)D and 1,25(OH)_2_D_3_. DHCR7 shunts vitamin D precursors toward cholesterol biosynthesis. VDR binds 1,25(OH)_2_D_3_ to activate gene transcription and regulates vitamin D metabolism. A potentially important clinical question is whether the polymorphism of these genes affects the vitamin D_3_ supplementation in diabetic patients with adequate vitamin D administration. In this study, we investigated the question in diabetic patients with long-term administration of vitamin D_3_ supplements.

## 2. Materials and Methods

### 2.1. Study Design and Participants

This study was a randomized controlled trial conducted at primary healthcare outpatient clinics in Lishui city (latitude: 28° N), which was located in Zhejiang Province, southeastern China. Individuals aged 50 years and older with a diagnosis of T2DM according to the diabetes diagnostic criteria set by the WHO in 1998 [22] were screened from residents in the local community in Lishui. The main exclusion criteria were as follows: impaired renal function (estimated glomerular filtration rate < 30 mL/min/1.73m^2^, calculated from serum creatinine using the modification of renal disease formula); hypercalcemia (serum calcium > 2.65 mmol/L) of any reason; urolithiasis; serum 25(OH)D > 60 ng/mL or calcitriol use; and major diseases including active cancer in the past five years, current acute inflammation, and other serious complications of diabetes. Withdrawal criteria for premature termination of the trial were as follows: serum 25(OH)D > 100 ng/mL, onset of hypercalcemia, hypersensitivity to cholecalciferol, and onset of urolithiasis.

**Figure 1:**
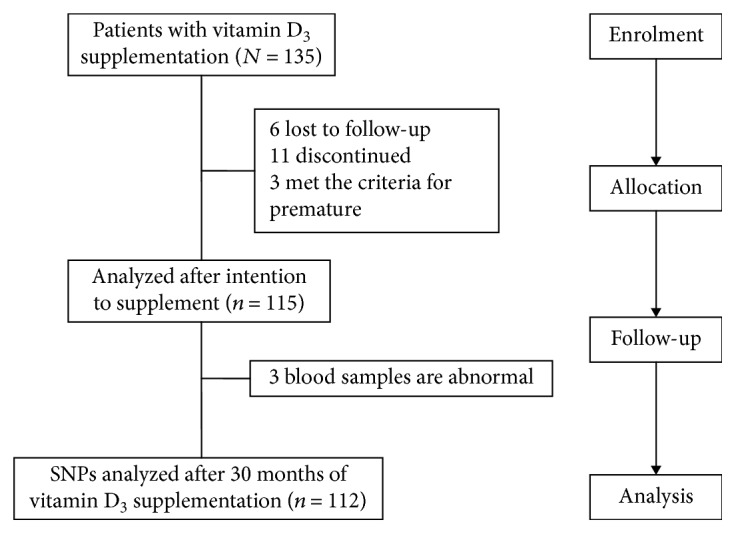
Flow diagram of study selection process.

Subjects received 800 IU vitamin D_3_ capsules (Xingsha Pharmaceuticals (Xiamen) Co., Ltd., China) that were taken daily at any time during the day and continued for a period of 30 months (from February 2015 to August 2017). A total of 115 diabetic patients were included in the study (Figure 1, flow diagram), and all participants provided written informed consent. This study conformed to the principles set by the Declaration of Helsinki and was approved by the Ethics Committee of Soochow University (ESCU-20160001).

### 2.2. Anthropometric Measurements

Before vitamin D_3_ supplementation, a standard questionnaire was used to collect information regarding participants' demographics (i.e., age, sex, and district), physical activity, diabetes treatment (oral antihyperglycemic agent, insulin, or neither), and past calcium supplementation.

Height and body weight were measured in light clothing, and body mass index (BMI) was defined as body weight divided by height squared (kg/m^2^). Waist circumference was measured midway between the iliac crest and the lowest rib and hip circumference over the great trochanters. Then, waist-to-hip ratio (WHR) was calculated. Blood pressure was measured by a digital sphygmomanometer (HEM-7211, Omron, Japan) three times while participants were in the relaxed sitting position after 15 min of rest. There is a 5 min rest period between each measurement, and the mean value of the three measurements were used for analysis. After a 5 min or longer rest, pulse rate was measured using pulse palpation over a 30 s period.

### 2.3. Biochemical Measurements

Fasting blood samples were collected for quantification of metabolic parameters. Participants were instructed to take all regular medications except for diabetes medication and take no aspirin or nonsteroidal anti-inflammatory drugs for 48 hours before the visit except those medications that were taken regularly. Peripheral venous blood sample was collected from each participant in a 10 h overnight fasting state for biochemical analysis. Serum 25(OH)D concentration was determined by chemiluminescence immunoassay using an automatic chemiluminescence immunoassay analyzer (ADVIA Centaur XP, Siemens Healthcare diagnostics Inc., Tarrytown NY, USA). The intra and interassay coefficients of variation were 5.2% and 7.2% at the mean level of 28.2 ng/mL, respectively. According to the Endocrine Society clinical practice guideline [23], vitamin D_3_ insufficiency and sufficiency were defined as serum 25(OH)D < 30 and ≥30 ng/mL. The concentrations of fasting blood glucose (FBG) and lipid profiles including total cholesterol (TC), triglyceride (TG), high-density lipoprotein cholesterol (HDL-C), and low-density lipoprotein cholesterol (LDL-C) were determined with the use of enzymatic method on an automatic biochemistry analyzer (COBAS c702; Roche Diagnostics GmbH, Mannheim, Germany). Fasting insulin (FINS) levels were measured with electrochemiluminescence immunoassay on COBAS e601 (Roche Diagnostics GmbH, Mannheim, Germany). The homeostatic model assessment of insulin resistance (HOMA-IR) was computed by using the formula: [FBG (mmol/L) × fasting insulin (mIU/L)]/22.5. The levels of glycosylated hemoglobin (HbA1c) and high-sensitive C-reactive protein (Hs-CRP) were measured by immunoturbidimetry method (Roche Diagnostics GmbH, Mannheim, Germany). Abnormal standards for indicators such as blood glucose and blood lipids reference prevention guide standards of care for type 2 diabetes in China [24] and guidelines for the prevention and treatment of dyslipidemia in Chinese adults [25]. All blood samples were analyzed under blinded conditions. The lab tests were repeated at baseline and after 6, 12, 18, and 30 months.

### 2.4. SNP Selection and Genotyping

Eight candidate SNPs from five vitamin D pathway genes (CYP2R1, CYP27B1, CYP24A1, DHCR7, and VDR) associated with 25(OH)D or other health outcomes were selected by reviewing the literature on vitamin D_3_ supplementation and metabolic gene correlation and the NCBI gene pool. The eight SNPs were CPY2R1 variant-rs10766197, CYP21R1 variant-rs 10741657, CYP27B1 variant-rs rs10877012, CYP27B1 variant-rs 4646536, CYP24A1 variant-rs 6013897, DHCR7 variant-rs 12785878, VDR variant-rs 2228570, and VDR variant-rs 1544410, respectively ([Supplementary-material supplementary-material-1]).

Genomic DNA was extracted from 250 *μ*L whole blood samples using Blood Genomic DNA Extraction Kit (Shanghai Jizhen Biological Technology Co., Ltd., Shanghai, China). The purity and concentration of Genomic DNA was detected by NanoDrop One Microvolume UV-vis spectrophotometer (Thermo Fisher Scientific, USA). And total DNA samples were diluted to the same concentration for downstream testing. Genotype analysis of key polymorphisms of vitamin D metabolic pathway was carried out using TaqMan^®^ probes (Applied Biosystems Foster City, CA); PCR reactions were performed in 10 *μ*L total volume, using 20 ng of DNA in TaqMan® Universal Master Mix with specific primers and probes (Applied Biosystems, Foster City, CA). The Bio-Rad CFX96 instrument equipped with the Bio-Rad CFX manager was used to assess the allelic content.

### 2.5. Statistical Analysis

Categorical variables were expressed as frequency (%), and continuous variables were expressed as mean ± standard deviation (SD). The serum 25(OH)D level of individuals after supplementation was represented by the average value of four-times tests during supplementation period. Chi-square or Fisher's exact test was used for the intergroup comparison of categorical variables. Paired *t*-test was used for the comparison of 25(OH)D level before and after supplementation. Chi-square was used to test whether the distribution of investigated SNPs meets Hardy-Weinberg equilibrium. Linkage disequilibrium test was measured using D′. The RRs for low level of 25(OH)D associated with various SNPs was estimated using log-binomial regression adjusted by age and outdoor activity. SNPStats software was used for Hardy-Weinberg equilibrium and linkage disequilibrium tests and SAS 9.3 software was used for other statistical analyses. All tests were two-side tests, and *P* < 0.05 was considered to be statistically significant.

## 3. Results

### 3.1. General Characteristics of the Subjects

A total of 112 patients with diabetes were included in the SNPs analysis, including 31 males and 81 females, respectively. Compared with before supplementation, the 25(OH)D level of each detection time point was significantly increased after supplementation (*P* < 0.05), and the mean value of the four tests after supplementation was 30.61 ± 5.04 ng/mL. At baseline, 17.86% of patients had 25(OH)D ≥ 30 ng/mL, while the percentage increased to 55.40% after supplementation, and 44.60% of patients still had a 25(OH)D level below 30 ng/mL (Table 1). Except CYP27B1 (rs10877012 and rs4646536), other investigated genotypes such as CPY2R1 (rs10766197 and rs10741657), CYP24A1 (rs6013897), DHCR7 (rs12785878), and VDR (rs2228570 and rs1544410) all met the Hardy-Weinberg equilibrium. Two SNPs, rs10877012 and rs4646536, showed complete linkage disequilibrium ([Supplementary-material supplementary-material-1]).

### 3.2. 25(OH)D Level and Insufficiency Risk in Patients with Different SNP Genotypes before and after Supplementation

Before supplementation, patients with different CYP24A1 (rs6013897) genotypes had different levels of serum 25(OH)D. All patients with A/A genotype had sufficient 25(OH)D. For other genes, different genotypes did not affect serum 25(OH)D level (*P* > 0.05) ([Supplementary-material supplementary-material-1]). After supplementation, the 25(OH)D levels in the patients with different CYP27B1 genotypes (rs10877012 and rs4646536) are significantly different (*P* < 0.05). The proportion of patients with rs10877012 G/T genotype to achieve ≥30 ng/mL was 71.79%, while the proportions in other two genotypes were only 40%-50%. Relative to T/T, the RR in patients with G/T of rs10877012 to experience 25(OH)D insufficiency (<30 ng/mL) was 0.544 (95% CI: 0.291-0.917); after adjusting age, sex, and outdoor activity, the RR was 0.560 (95% CI: 0.292-0.970). For other genes, the 25(OH)D levels in the patients with different genotypes were not significantly different (*P* > 0.05) (Table 2).

### 3.3. Changes of 25(OH)D Levels in Patients with Different Genotypes of CYP27B1 (rs10877012) before and after Supplementation

**Figure 2:**
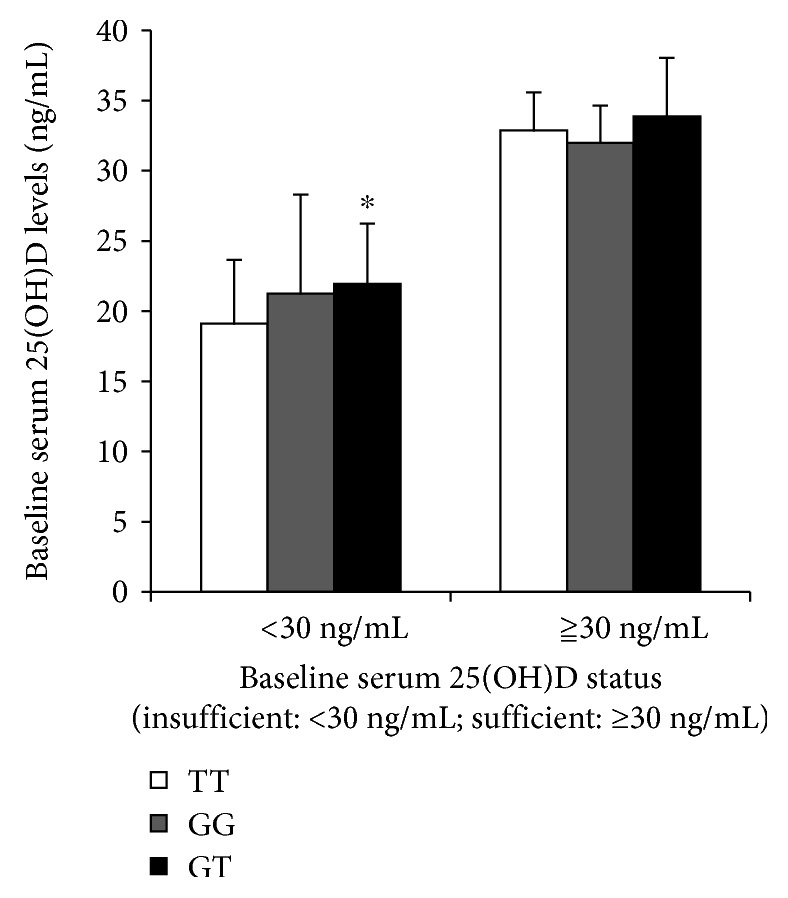
The levels of serum 25(OH)D in patients with different CYP27B1 (10877012) genotypes before vitamin D_3_ supplementation. White bars: CYP27B1 rs10877012 TT genotype; gray bars: CYP27B1 rs10877012 GG genotype; black bars: CYP27B1 rs10877012 GT genotype. All data were expressed as means ± SD. ^∗^*P* < 0.05.

**Figure 3:**
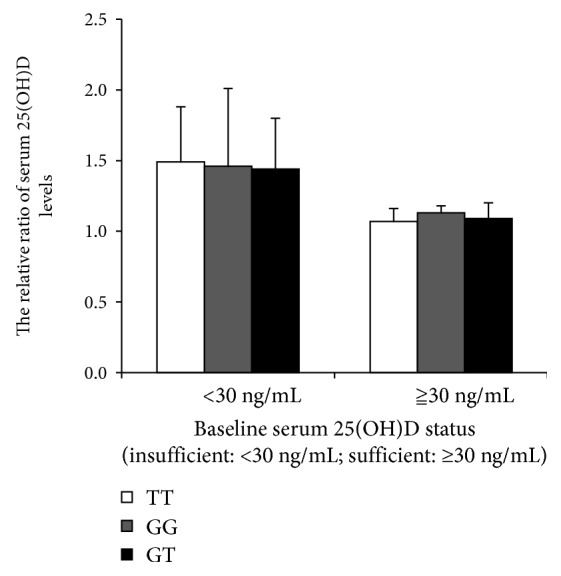
Comparison of serum 25(OH)D in the patients with different CYP27B1 rs10877012 genotypes before and after vitamin D_3_ supplementation. The vertical axis is the relative ratio of serum 25(OH)D before and after 30 months of vitamin D3 supplementation. Insufficiency: serum 25(OH)D level of <30 ng/mL; sufficiency: serum 25(OH)D level of ≥30 ng/mL. There was no significant difference in the increase of serum 25(OH)D levels in patients of TT, GG, and GT genotype after supplementation. All *P* values were >0.05.

There were significant differences in the 25(OH)D levels of TT, GG, and GT genotype in patients of baseline 25(OH)D < 30 ng/mL (*P* = 0.045), and patients with GT was significantly higher than patients with TT (*P* = 0.017). While there were no significant differences in the 25(OH)D levels of the three genotypes in patients with baseline 25(OH)D ≥ 30 ng/mL (Figure 2), in patients with baseline 25(OH)D < 30 ng/mL and ≥30 ng/mL, there was no significant difference in the increase of serum 25(OH)D levels after vitamin D_3_ supplementation (Figure 3).

### 3.4. 25(OH)D Level and Insufficiency Risk in Patients with Different CYP27B1 (10877012) Genotypes after Stratification of Vitamin D Metabolism-Related Indicators

Relative to T/T, after adjusting age and outdoor activity, the RR of 25(OH)D insufficiency (<30 ng/mL) in female patients with CYP27B1 (rs10877012) G/T type was 0.423 (95% CI: 0.186-0.820); the RR of 25(OH)D insufficiency (<30 ng/mL) in overweight (BMI ≥ 24) patients with CYP27B1 (rs10877012) G/T type was 0.449 (95% CI: 0.312-0.809); the RR of 25(OH)D insufficiency (<30 ng/mL) in patients with abnormal total cholesterol (TC ≥ 5.20 mmol/L) with CYP27B1 (rs10877012) G/T type was 0.384 (95% CI: 0.087-0.494) ([Supplementary-material supplementary-material-1]).

## 4. Discussion

Vitamin D deficiency and insufficiency has been involved in the development of diabetes. It gains much attention that whether vitamin D_3_ supplementation is affected by genetic variation in key genes associated with vitamin D metabolism. In this study, we found that the SNPs of vitamin D metabolism-related genes rs10877012 and rs4646536 (completely linked) were correlated with serum 25(OH)D concentration after long-term adequate supplementation of 800 IU/day vitamin D_3_ per day for 30 months in diabetic patients. In patients with insufficient 25(OH)D levels at baseline, the 25(OH)D level of patients with rs10877012GT genotype was significantly higher than that of TT, but there was no significant difference in the proportion of increase after vitamin D_3_ supplementation. Moreover, 71.79% patients carrying G/T genotype of rs10877012 achieved sufficient 25(OH)D levels (≥30 ng/mL), showing a better response to vitamin D_3_ supplementation than those carrying T/T or G/G genotype. We found the phenomenon that heterozygote variants of rs10877012 are related to the effect of vitamin D supplementation through statistical analysis and not also one of the homozygotes. Similar to our results, Orton et al. reported that the associations of the heterozygous genotype CYP27B1 rs4646536 (C/T) with 25(OH)D level in a Canadian multiple sclerosis study [26]. However, the pattern of association between the alleles and 25OHD is not consistent in the other studies [21]. The inconsistency of this association may be due to the relatively small sample sizes in these studies or the different ethnicities.

The guideline of the Institute of Medicine (IOM) recommends an increase of vitamin D_3_ intake of 200 IU/day compared with the guideline of 1997 [27]. Adults less than 70 years old need to meet a daily intake of 600 IU through recommended diets. For those above 70 years old, 800 IU is required per day [27]. IOM also increased the maximum safe dose of vitamin D_3_ supplementation to 4000 IU per day [27].The supplementation dose used in our experiments was 800 IU/day which was considered to be sufficient. Our study suggested that after long-term supplementation, there was still about 50% patients whose 25(OH)D level did not reach the “ideal level” accepted by current standards (≥30 ng/mL). Genetic polymorphism affects patient's response to adequate vitamin D_3_ supplementation; therefore, it is inappropriate to define a unique “ideal level” for peoples with different genetic backgrounds, e.g., for the diabetic patients carrying rs10877012 T/T and G/G genotype, ideal level of serum 25(OH)D may be lower than other SNPs. On the contrary, for diabetic patients with rs10877012 T/T or G/G genotype, especially women, overweight, and abnormally high-total cholesterol diabetes, vitamin D supplementation dose should be higher than that of the routine supplementary dose. These findings increase our understanding of vitamin D homeostasis and may help determine the benefit/risk of vitamin D_3_ supplementation in Chinese diabetic patients.

Previous genome-wide association studies (GWAS) identified several SNPs in the vitamin D metabolic pathway associated with serum 25(OH)D status [20, 21]. However, few studies have investigated whether common genetic variants affect the response to vitamin D_3_ supplementation. Several studies in a nondiabetic population have found rs10766197 (CYP2R1) [28], rs10741657 (CYP2R1) [29, 30], rs6013897 (CYP24A1) [29], rs2282679 (GC) [31], and rs7968585 (VDR) [29] were associated with the increase of serum 25(OH)D after vitamin D_3_ supplementation. Similar studies were also conducted in gestational diabetes patients [32], but studies in patients with type 2 diabetes have not been reported. Our study found that although genetic variation of CYP27B1 was not significantly associated with baseline 25(OH)D level, it was associated with the response to vitamin D_3_ supplementation. The published GWAS studies [20, 21] found that SNPs affecting baseline 25(OH)D were different from SNPs affecting the response to cholecalciferol supplementation, which was in line with our study. However, SNPs which have been reported to be associated with 25(OH)D in population cross-sectional and longitudinal studies, including DHCR7 (rs12785878) [33], CYP2R1 (rs10766197 and 10741657) [28, 34], CYP2 24A1 (rs6013897) [33, 35], and VDR (rs2228570 and 1544410) [26, 36], were not found to be associated with baseline 25(OH)D level or response to vitamin D_3_ supplementation in our study. Further studies will elucidate effects of these SNPs on vitamin D metabolism.

CYP27B1 encodes 1a-hydroxylase that converts 25(OH)D to its active form, 1,25(OH)_2_D_3_. CYP27B1 is a cytochrome P450 most strongly associated with the vitamin D status. The SNP rs10877012 that resides at position 1260 of CYP27B1 was widely explored for the association with 25(OH)D [19]. In a gestational diabetes study [37] and a large-scale cohort study [38], the C allele of rs10877012 was associated with lower level of 25(OH)D. The association of the rs10877012 C allele with low 25(OH)D level was also reported in an African-American study [39]. Although the effect of rs10877012 on 25(OH)D level has been verified in candidate gene studies, data regarding how this SNP regulates serum 25(OH)D is limited. Our study suggests that rs10877012 (CYP27B1) does alter the response of diabetic patients to vitamin D_3_ supplementation. These results indicated that different mechanisms regulate 25(OH)D levels from vitamin D_3_ supplementation. CYP27B1 functions downstream of circulating 25(OH)D; therefore, rs10877012 may alter the role of CYP27B1 in the metabolic feedback loop or regulate the metabolic rate of 25(OH)D [26]. The gene variant may reduce the efficiency of 25(OH) D hydroxylation to 1,25-dihydroxy vitamin D (1,25-(OH)2D), which may lead to a phenomenon that if the condition of 25(OH)D level is normal or even higher, 1,25(OH)_2_D_3_ level in serum is low. In other words, some people may have mild “vitamin D resistance” but lack of the rickets displays.

The present study was a population-based and prospective study. The internal effectiveness was maximized by using uniform regimen and dosage of vitamin D_3_ supplementation, and minimizing participant's variation. Serum 25(OH)D was measured every 6 months to eliminate seasonal effect and increase reproducibility. We used rigorous statistical methods to examine the interaction between genotype and vitamin D_3_ supplementation, minimizing the effect of confounding factors on the results. Finally, we focused on candidate SNPs which are previously reported to be associated with 25(OH)D or other health outcomes, covering five key genes involved in the vitamin D metabolism. However, some limitations of the study are also worth mentioning. First, the relatively small sample size of our study may affect the results. It is necessary to expand the sample size to conduct more comprehensive analyses. Second, we did not examine the “downstream” markers for vitamin D status because 25(OH)D concentration is considered the most reliable indicator of vitamin D status. Other molecules, such as 1,25-(OH)2D or parathyroid hormone, have greater intraindividual variability and are affected by other factors in addition to vitamin D status. Finally, we only studied diabetic patients in the Chinese population. It is unclear whether the genetic variations found in this study affect the vitamin D status in other ethnic populations or people with other diseases, which deserves further study.

## 5. Conclusions

In conclusion, we systematically studied the association between single nucleotide polymorphisms of vitamin D metabolism-associated genes and the change of serum 25(OH)D level after long-term vitamin D_3_ supplementation in patients with type 2 diabetes. We found that CYP27B1 variation might be a predictive factor for vitamin D insufficiency after vitamin D_3_ supplementation. Our study suggests that SNP analysis can identify high-risk diabetic patients who may require higher doses of vitamin D_3_ supplementation, and vitamin D_3_ supplementation should be personalized.

## Figures and Tables

**Table 1:** Basic characteristics of diabetic patients.

Variable	Value (*n* (%) or mean ± SD)
Demographic indicators	
Sex (*n*, %)	
Male	31 (27.7)
Female	81 (72.3)
Age (y), mean ± SD	66.3 ± 9.1
Physical activity (h/day), *n* (%)	
<1 h	50 (44.6)
≥1 h	61 (55.4)
Calcium supplementation	
Yes	24 (21.4)
No	88 (78.6)
Region (*n*, %)	
Urban	48 (42.9)
Rural	64 (57.1)
Anthropometry	
Height (cm)	156.9 ± 6.7
Weight (kg)	61.5 ± 11.0
BMI (kg/m^2^)	24.9 ± 3.9
Waist (cm)	85.8 ± 10.6
Hipline (cm)	95.7 ± 9.3
Waist-hipline ratio (cm/cm)	0.9 ± 0.1
Pulse (time/min)	75.8 ± 6.9
Systolic blood pressure (mmHg)	138.5 ± 15.9
Diastolic blood pressure (mmHg)	80.1 ± 8.6
Supplementary phase	25(OH)D (ng/mL)
Before supplementation	22.7 ± 6.9
Supplement for 6 months	29.2 ± 6.8^∗^
Supplement for 12 months	29.0 ± 5.4^∗^
Supplement for 18 months	28.9 ± 4.6^∗^
Supplement for 30 months	35.3 ± 7.2^∗^
Mean after supplementation	30.6 ± 5.0^∗^

^∗^Compared with before supplementation, *p* < 0.05.

**Table 2:** 25(OH)D level and insufficiency risk in patients with different SNP genotypes after vitamin D_3_ supplementation.

Gen	SNP	Gene locus	25(OH)D ≥ 30 ng/mL, *n* (%)	25(OH)D < 30 ng/mL, *n* (%)	RR (95% CI)^a^	*P* ^b^	RR (95% CI)^c^	*P* ^d^
CYP2R1	rs10766197	G/G	30 (60)	20 (40)	1	0.666	1	0.693
		A/A	8 (50)	8 (50)	1.25 (0.63, 2.18)		1.19 (0.60, 2.10)	
		A/G	24 (52.17)	22 (47.83)	1.19 (0.76, 1.91)		1.202 (0.76, 1.92)	
	rs10741657	G/G	22 (48.89)	23 (51.11)	1	0.244	1	0.294
		A/A	11 (73.33)	4 (26.67)	0.52 (0.17, 1.10)		0.54 (0.18, 1.15)	
		A/G	29 (55.77)	23 (44.23)	0.87 (0.56, 1.33)		0.87 (0.56, 1.32)	

CYP27B1	rs10877012	T/T	26 (48.15)	28 (51.85)	1	**0.031**	1	**0.046**
		G/G	8 (42.11)	11 (57.89)	1.12 (0.66, 1.71)		1.15 (0.67, 1.81)	
		G/T	28 (71.79)	11 (28.21)	**0.54 (0.29, 0.92)**		**0.56 (0.29, 0.98)**	
	rs4646536	C/C	26 (48.15)	28 (51.85)	1	**0.031**	1	**0.046**
		T/T	8 (42.11)	11 (57.89)	1.12 (0.66, 1.71)		1.15 (0.67, 1.81)	
		C/T	28 (71.79)	11 (28.21)	**0.54 (0.29, 0.92)**		**0.56 (0.29, 0.98)**	

CYP24A1	rs6013897	T/T	43 (57.33)	32 (42.67)	1	0.209	1	0.212
		A/A	2 (100)	0 (0)	—		—	
		A/T	17 (48.57)	18 (51.43)	—		—	

DHCR7	rs12785878	T/T	18 (60)	12 (40)	1	0.082	1	0.110
		G/G	23 (67.65)	11 (32.35)	0.81 (0.41, 1.58)		0.82 (0.41, 1.60)	
		G/T	21 (43.75)	27 (56.25)	1.41 (0.88, 2.47)		1.39 (0.87, 2.45)	

VDR	rs2228570	T/T	14 (53.85)	12 (46.15)	1	0.964	1	0.943
		C/T	11 (57.89)	8 (42.11)	0.97 (0.61, 1.68)		1.02 (0.64, 1.76)	
		C/C	37 (55.22)	30 (44.78)	0.91 (0.435, 1.77)		0.92 (0.44, 1.78)	
	rs1544410	G/G	5 (71.43)	2 (28.57)	1	0.367	1	0.404
		A/G	57 (54.29)	48 (45.71)	0.63 (0.12, 1.48)		0.66 (0.12, 1.52)	

Note: RR of 25(OH)D < 30 ng/mL. ^a^Uncorrected RR. ^c^Corrects age and amount of outdoor activity. *P* value is 25(OH)D < 30 ng/mL versus ≥30 ng/mL in comparison of all SNP genotypes. ^b^Uncorrected RR. ^d^Corrects age and amount of outdoor activity. Bold values are *p* < 0.05.

## Data Availability

The data used to support the findings of this study are currently under embargo while the research findings are commercialized. Requests for data, 12 months after publication of this article, will be considered by the corresponding author.

## Supplementary Materials

Supplementary Materials Table S1: the description of candidate SNPs genotyped. Table S2: a description of baseline 25(OH)D levels in patients with different SNP genotypes, which the RRs for low level of 25(OH)D associated with various SNPs was re-estimated using log-binomial regression. Table S3: a description of 25(OH)D level and insufficiency risk in patients with different CYP27B1 (10877012) genotypes after stratification of vitamin D metabolism-related indicators (sex, BMI, and TC), which the RRs for low level of 25(OH)D associated with various SNPs were re-estimated using log-binomial regression.
