# Supplementary material for: The Association between Polymorphisms of Vitamin D Metabolic-Related Genes and Vitamin D3 Supplementation in Type 2 Diabetic Patients
Source: J Diabetes Res. 2019 Sep 8;2019:8289741. doi: 10.1155/2019/8289741 (PMC6754883; doi:10.1155/2019/8289741)
Supplement: Supplementary Materials — Table S1: the description of candidate SNPs genotyped. Table S2: a description of baseline 25(OH)D levels in patients with different SNP genotypes, which the RRs for low level of 25(OH)D associated with various SNPs was re-estimated using log-binomial regression. Table S3: a description of 25(OH)D level and insufficiency risk in patients with different CYP27B1 (10877012) genotypes after stratification of vitamin D metabolism-related indicators (sex, BMI, and TC), which the RRs for low level of 25(OH)D associated with various SNPs were re-estimated using log-binomial regression. [file 8289741.f1.pdf]

Table S1. Description of Candidate SNPs Genotyped

LD [a]	SNP [b]	Gene	SNP location or coding change	Alleles	N [c]	MAF
	rs10766197	CPY2R1 (25-hydroxylase)	5’	G/A	112	0.46
	rs 10741657		5’	G/A	112	0.40
*	rs10877012	CYP27B1 (1- hydroxylase)	5’	G/T	112	0.33
*	rs 4646536		intron	T/C	112	0.33
	rs 6013897	CYP24A1 (24- hydroxylase)	3’	T/ A	112	0.20
	rs12785878	DHCR7 (7-dehydro-cholesterol reductase)[d]	5’	T/ G	112	0.26
	rs2228570	VDR (Vitamin D receptor)	Start codon [e]	C/T	112	0.37
	rs1544410		intron	G/A	112	0.39

Abbreviations: LD, linkage disequilibrium; SNP, single nucleotide polymorphism; MAF, minor allele frequency.

[a] Within each gene, * (or ^) indicates SNPs in high LD with each other (r^2>95%).

[b] VDR restriction fragment length polymorphisms: rs2228570 (fok1), rs1544410 (bsm1).

[c] Number of participants with genotype data available for analysis.

[d] DHCR7 SNPs (rs12785878) are located within introns in the upstream NADSYN1 gene.

[e] SNP rs2228570 (fok1) leads to new start site 9 bp upstream and VDR protein longer by 3 amino acids.

Table S2. **Baseline 25(OH)D levels in patients with different SNP genotypes**

Gen	SNP	Gene locus	25(OH)D≥30ng/mL, n (%)	25(OH)D<30ng/mL, n (%)	RR (95% CI) [a]	*P* [b]	RR (95% CI) [c]	*P* [d]
CPY2R1	rs10766197	G/G	8(16)	42(84)	1	0.363	1	0.116
		A/A	5(31.25)	11(68.75)	0.82 (0.52,1.09)		0.74 (0.60,0.99)	
		A/G	7(15.22)	39(84.78)	1.01 (0.84,1.21)		0.96(0.95,1.17)	
	rs10741657	G/G	8(17.78)	37(82.22)	1	0.246	1	0.388
		A/A	5(33.33)	10(66.67)	0.81 (0.49,1.11)		0.83 (0.51,1.14)	
		A/G	7(13.46)	45(86.54)	1.05 (0.88,1.27)		1.04(0.87,1.27)	
CPY27B1	rs10877012	T/T	9(16.67)	45(83.33)	1	0.865	1	0.803
		G/G	3(15.79)	16(84.21)	1.01 (0.76,1.24)		1.09(0.80,1.10)	
		G/T	8(20.51)	31(79.49)	0.95(0.76,1.16)		1.04 (0.80,1.06)	
	rs4646536	T/T	3(15.79)	16(84.21)	1	0.865	1	0.803
		C/C	9(16.67)	45(83.33)	0.99(0.81,1.33)		0.92 (0.91,1.25)	
		C/T	8(20.51)	31(79.49)	0.94(0.74,1.28)		0.95(0.93,1.28)	
CPY24A1	rs6013897	T/T	15(20.00)	60(80.00)	1	0.008	1	0.006
		A/A	2(100.00)	0(0.00)	-		0.03 (0.01,0.08)	
		A/T	3(8.57)	32(91.43)	1.14(0.98,1.33)		1.14 (0.98,1.19)	

Gen	SNP	Gene locus	25(OH)D≥30ng/mL, n (%)	25(OH)D<30ng/mL, n (%)	RR (95% CI) [a]	*P* [b]	RR (95% CI) [c]	*P* [d]
DHCR7	rs12785878	T/T	6(20.00)	24(80.00)	1	0.516	1	0.265
		G/G	4(11.76)	30(88.24)	1.10 (0.89,1.42)		1.11(0.95,1.14)	
		G/T	10(20.83)	38(79.17)	0.99(0.79,1.28)		0.96(0.92,1.22)	
VDR	rs2228570	T/T	4(15.38)	22(84.62)	1	0.871	1	0.972
		C/T	13(19.40)	54(80.60)	0.95 (0.79, 1.21)		0.99(0.81, 1.27)	
		C/C	3(15.79)	16(84.21)	0.99 (0.74, 1.29)		1.02 (0.75, 1.23)	
	rs1544410	G/G	20(19.05)	85(80.95)	1	0.113	1	0.255
		A/G	0(0.00)	7(100.00)	-	-	-	

Note: RR of 25(OH)D <30ng/mL, a: uncorrected RR, c: corrects age and amount of outdoor activity. *P* value is 25(OH)D <30ng/mL vs ≥30 ng/mL comparison in all SNP genotypes, b: uncorrected RR, d: corrects age and amount of outdoor activity.

Table S3. 25(OH)D level and insufficiency risk in patients with different CYP27B1(10877012)genotypes after stratification of vitamin D metabolism related indicators

index		genotype	25(OH)D ≥30 ng/mL, n (%)	25(OH)D<30 ng/mL, n (%)	RR (95%CI) [a]	*P* [b]	RR (95%CI) [c]	*P* [d]
Sex (female, male)								
	female	T/T	15(35.71)	23(58.97)	1	0.009	1	0.016
		G/G	6(14.29)	9(23.08)	0.99(0.56,1.55)		1.01(0.55,1.63)	
		G/T	21(50.00)	7(17.95)	0.41(0.19,0.77)		0.42 (0.19, 0.82)	
	male	T/T	11(55.00)	5(45.45)	1	0.785	1	0.590
		G/G	2(10.00)	2(18.18)	1.60(0.310,5.04)		2.35 (0.38, 9.41)	
		G/T	7(35.00)	4(36.36)	1.16(0.36,3.52)		1.49 (0.39, 4.58)	
BMI(≥24,<24)								
	≥24	T/T	9(30.00)	20(57.14)	1	0.031	1	0.025
		G/G	5(16.67)	7(20.00)	0.85 (0.43, 1.36)		0.79 (0.44, 1.31)	
		G/T	16(53.33)	8(22.86)	0.48(0.24,0.84)		0.45 (0.31, 0.81)	
	<24	T/T	17(53.13)	8(53.33)	1	0.229	1	0.229
		G/G	3(9.38)	4(26.67)	1.79 (0.64,4.15)		1.85 (0.63,3.65)	
		G/T	12(37.50)	3(20.00)	0.63 (0.15,1.80)		0.65 (0.15,2.08)	

TC(≥5.20 mmol/L,<5.20 mmol/L)								
	≥5.2mmol/L	T/T	9(31.03)	11(57.89)	1	0.002	1	0.013
		G/G	3(10.34)	6(31.58)	1.21 (0.58, 2.22)		1.52 (0.74, 3.86)	
		G/T	17(58.62)	2(10.53)	0.19(0.03,0.59)		0.38 (0.09, 0.49)	
	<5.2mmol/L	T/T	17(51.52)	17(54.84)	1	0.933	1	0.921
		G/G	5(15.15)	5(16.13)	1.00(0.42, 1.85)		0.98 (0.41, 1.81)	
		G/T	11(33.33)	9(29.03)	0.90(0.46, 1.58)		0.89(0.46, 1.55)	

Note: RR of 25(OH)D <30 ng/mL, a: uncorrected RR, c: corrects age and amount of outdoor activity. *P* value is 25(OH)D<30 ng/mL vs ≥30 ng/mL comparison in all SNP types, b: uncorrected RR, d: corrects age and amount of outdoor activity.
